# Crop Establishment Practices Are a Driver of the Plant Microbiota in Winter Oilseed Rape (*Brassica napus*)

**DOI:** 10.3389/fmicb.2017.01489

**Published:** 2017-08-09

**Authors:** Ridhdhi Rathore, David N. Dowling, Patrick D. Forristal, John Spink, Paul D. Cotter, Davide Bulgarelli, Kieran J. Germaine

**Affiliations:** ^1^Envirocore, Dargan Research Centre, Institute of Technology Carlow, Ireland; ^2^Teagasc Crops Research Centre Carlow, Ireland; ^3^Teagasc Food Research Centre, Moorepark, Fermoy, and the APC Microbiome Institute Cork, Ireland; ^4^Plant Sciences, School of Life Sciences, University of Dundee at the James Hutton Institute Invergowrie, Scotland

**Keywords:** tillage, oilseed rape, microbiota, next generation sequencing, 16S rRNA gene

## Abstract

Gaining a greater understanding of the plant microbiota and its interactions with its host plant heralds a new era of scientific discovery in agriculture. Different agricultural management practices influence soil microbial populations by changing a soil’s physical, chemical and biological properties. However, the impact of these practices on the microbiota associated with economically important crops such as oilseed rape, are still understudied. In this work we investigated the impact of two contrasting crop establishment practices, conventional (plow based) and conservation (strip–tillage) systems, on the microbiota inhabiting different plant microhabitats, namely rhizosphere, root and shoot, of winter oilseed rape under Irish agronomic conditions. Illumina 16S rRNA gene sequence profiling showed that the plant associated microhabitats (root and shoot), are dominated by members of the bacterial phyla *Proteobacteria, Actinobacteria* and *Bacteroidetes*. The root and shoot associated bacterial communities displayed markedly distinct profiles as a result of tillage practices. We observed a very limited ‘rhizosphere effect’ in the root zone of WOSR, i.e., there was little or no increase in bacterial community richness and abundance in the WOSR rhizosphere compared to the bulk soil. The two tillage systems investigated did not appear to lead to any major long term differences on the bulk soil or rhizosphere bacterial communities. Our data suggests that the WOSR root and shoot microbiota can be impacted by management practices and is an important mechanism that could allow us to understand how plants respond to different management practices and environments.

## Introduction

Soil is the foundation of productive agriculture and represents the most diverse and significant ecosystem on Earth (Roger-Estrade et al., 2010). The collective microbial community in soil, referred to as the microbiota, underpins many soil ecosystem functions (Kibblewhite et al., 2008) regulating soil fertility, biogeochemical cycling and impacting on plant performance (Fierer et al., 2012). For instance, the host plant is assisted by its microbiota in nutrient acquisition, phytohormone production, phytotoxic compound degradation, tolerance to biotic and abiotic stress and the suppression of pathogens (Whipps, 2001; Compant et al., 2005; Glick, 2012). In return, the plant provides a favorable environment for microbial growth and a continuous supply of carbon rich rhizodeposition (Zhang et al., 2009). Consequently, and similar to other eukaryotic organisms, plants can be considered holobionts whose growth, development and health are ultimately determined by the outcome of host–microbiota interactions (Bordenstein and Theis, 2015). In this respect, many research studies have shown that plant–microbe interactions are not only essential for developing a understanding of plant growth and health, but are of considerable importance with respect to developing sustainable agricultural practices (Berg et al., 2014).

Agricultural management practices influence soil physical, chemical, and biological properties, which have direct impacts on soil microbial composition and behavior (Jangid et al., 2008). Conventional tillage (CT) practices invert the soil to a depth of about 20–35 cm through plowing, and leave < 30% of crop residues on the soil surface. The mechanical disruption of soil leads to water and nutrient losses, soil erosion, soil degradation due to low organic matter content and a fragile soil structure (Vian et al., 2009). Shifting CT to conservation tillage practices such as strip tillage (ST), significantly reduces these impacts (Hobbs et al., 2008). Conservation tillage generally encompasses shallow working depths without soil inversion and retains >30% of crop residues on soil surface which, over a number of years, helps to maintain soil moisture, increases soil organic matter content, reduces soil erosion, promotes soil fertility and biological activity (Vian et al., 2009). However, in oceanic /temperate regions, conservation tillage presents challenges from a weed control perspective and crop establishment can be difficult in wetter conditions with slower early crop growth.

Tillage practices have been shown to influence microbial community structure, taxonomic composition, microbial abundance and activity by changing the physicochemical properties of soil (García-Orenes et al., 2013). For instance, Zhang et al. (2012) reported that microbial biomass accumulation was tillage dependent and recommended conservation tillage as an effective component to improve soil quality and sustainability. Smith et al. (2016) showed that there was a significant difference in the soil microbial community structure and predicted function as a consequence of CT or no-tillage systems. For instance, bacterial populations carrying genes involved in protein degradation, ammonia assimilation and denitrification were higher in the no-tillage system, while bacterial populations carrying genes involved in ammonification and nitrous oxide production were higher in conventional tilled soils. Zhang et al. (2014) showed that phospholipid fatty acid (PLFA) profiles and soil enzyme activities were significantly higher in no tilled soils than in ridge tilled soils.

Oilseed rape (*Brassica napus* L.) is the world’s third largest source of vegetable oil (USDA-FAS, 2015) used in human nutrition and as a source of oil for biodiesel production. Oilseed rape (OSR) is grown as spring oilseed rape (SOSR) and winter oilseed rape (WOSR) varieties. WOSR is also an important break crop in cereal crop rotation and can significantly reduce the rate of ‘take-all’ fungal disease (*Gaeumannomyces graminis* var. *tritici*) as a result, improves the yield of subsequent cereal crops (Angus et al., 1991; Hilton et al., 2013). Although several studies provided insights into host–microbiota interactions in OSR (Germida et al., 1998; Macrae et al., 2000; Kaiser et al., 2001; Hilton et al., 2013), they generally utilized low-resolution, analytical protocols which make it difficult to develop a fundamental understanding of the significance of these microbes to OSR production. For instance, the impact of soil tillage on the microbiota of OSR, and the potential implications for crop production, remains largely unknown.

The aim of this research was to obtain detailed knowledge of (a) the composition of the bacterial microbiota associated with WOSR and (b) how this composition is influenced by conventional plow (CT) and conservational strip tillage (ST) practices. In particular, we were motivated to test the hypothesis that different WOSR microhabitats (rhizosphere, root and shoot) host distinct microbiota whose composition is modulated by tillage practices.

## Materials and Methods

### Experimental Design

The plant and soil samples for this study were taken from a field experiment evaluating the effect of crop establishment systems on the growth and development of WOSR. The establishment systems comprised of: (1) a conventional plow based system (CT) and (2) a low-disturbance conservation ST system. The conventional establishment system comprised of mouldboard plowing which inverted the soil to a depth of 230 mm, 2 days prior to sowing. The plowed soil then received secondary plowing to 100 mm depth with a rotary power harrow and the WOSR was sown at 10 mm depth at row spacing of 125 mm using a conventional mechanical delivery seed drill operated in combination with the power harrow. The ST establishment system deployed was a non-inversion system, comprised of a single cultivation/seeding pass of a rigid leg cultivator with legs spaced at 600 mm apart which were operated at 200 mm depth. These forward facing tines, with side ‘wings’ giving additional soil disturbance, worked directly in the cereal residue of the previous crop, disturbing approximately 50% of the surface width between the legs. This was the first year that ST was used in this field, as in previous years plow based tillage practices had been used. Seeding was by metered pneumatic delivery of seed to a point behind the cultivator leg, giving a row spacing of 600 mm. For the microbiota studies, plant and soil samples were taken from these two establishment systems (CT and ST) in three replicated plots. The trial was a randomized block design with individual plot dimensions of 24 m × 4.8 m and was located in an area known locally as the sawmill field at the Teagasc Crops Research Centre, Oak Park, Carlow, Ireland (52.857478°N, -6.922467°W). The previous crop was winter barley and cereal crops had been sown for more than 5 years previously. The WOSR variety ‘Compass’ was sown at a seed-rate of 60 seeds/m^2^ on 28th August 2013 in both establishment systems. Subsequent to seeding, the soil surface was rolled using a ring roller. The top soil was a well-drained sandy loam overlying inter-bedded layers of sand, gravel and silt/clay. The top soil had a sand content of 50–70% with less than 20% clay. Physical and chemical characterization of the soil substrates used in this study described in Supplementary Table S1. Crop management, other than crop establishment, followed standard practices for WOSR production in this region. A pre-emergence selective herbicide (quinmerac and metazachlor) was applied post seeding for weed control. The crop received two fungicide applications (prothioconazole) in October and March for phoma stem canker (*Leptosphaeria* sp.) and light leaf spot (*Pyrenopeziza brassicae*) control. Phosphate (P) and potassium (K) fertilizer was applied on the basis of soil test results post sowing according to Teagasc guidelines (Coulter and Lalor, 2008). Fertilizer N (a total of 225 kg N/ha) was applied in three equal applications at 2 week intervals starting in late February.

### Sample Collection of Bulk Soil, Rhizosphere and Plant Fractions

Bulk soil and plant samples were collected from the two treatments; CT and conservation ST in triplicate from three replicate plots per treatment at the harvesting stage (∼330 days after sowing). Bulk soil samples were collected from a depth of 0–25 cm, in triplicate from the edges of each plot, using a hand auger. For each plot, composite soil samples were prepared by thoroughly mixing the triplicate samples and a representative subsample of this was collected in sterile 50 mL Falcon tubes. The plant samples were processed into three plant microhabitat zones i.e., rhizosphere soil, root and shoot. The excess soil from the root was removed by manual shaking, leaving ∼1 mm of rhizosphere soil still attached to the root. The rhizosphere soil attached to the root was scraped off with a sterile forceps into sterile 50 mL Falcon tube. The root samples were washed separately in 50 mL Falcon tubes containing 30 mL of Phosphate Buffered Saline (130 mM NaCl, 7 mM Na_2_HPO_4_, 3 mM NaH_2_PO_4_, 7.0 pH, 0.02 % Silwet L-77) to remove the tightly adhered microbes from the root surface followed by a sonication step (30 s at 50–60 Hz) as described by Lundberg et al. (2012). Shoot samples were not surface sterilized to make sure that both endophytic and epiphytic communities could be recovered. Root and shoot samples were frozen using liquid nitrogen and stored in pre-labeled sterile 50 mL Falcon tubes. All the samples were stored in -80°C until required for DNA extraction.

### DNA Extraction from Soil and Plant Microhabitat Zones

DNA extractions were performed on 3 bulk soil, 3 rhizosphere soil, 3 root and 3 shoot samples per plot with 3 plots per treatment (CT and ST). For DNA extraction in soil, 0.25 g of soil was taken individually from each composite soil sample and processed according to the protocol from MoBio PowerSoil^TM^ DNA isolation kit (Carlsbad, CA, United States). Total soil DNA was eluted in 50 μL of sterile water (Sigma–Aldrich). For DNA extraction from the plant samples, 0.5 g of plant tissues were individually ground in liquid nitrogen. The DNA was extracted following 2% cetyl trimethylammonium bromide (CTAB) method described by Doyle (1990). Total plant DNA was eluted in 100 μL of sterile water. Concentration and purity of DNA was determined by Nanodrop spectrophotometry (Thermo Scientific, Wilmington, DE, United States). Post quantification, all DNA samples were normalized to 10 ng/μL. The three DNA samples from each microhabitat zone per block were pooled (e.g., the three DNA samples from the shoot samples from block 1 were pooled) to give representative DNA samples of bulk soil, rhizosphere, root and shoot from each block.

### Illumina Sequencing of 16S rRNA Gene Amplicon Libraries

The amplicon library of bacterial DNA was generated using the PCR primers:

341F (5′-TCGTCGGCAGCGTCAGATGTGTATAAGAGACAGCCTACGGGNGGCWGCAG-3′), 785R (5′-GTCTCGTGGGCTCGGAGATGTGTATAAGAGACAGGACTACHVGGGTATCTAATCC-3′), with Illumina adapter overhang sequences (underlined) which covered ∼464 bp of the hypervariable regions V3 and V4 of the 16S rRNA gene (Klindworth et al., 2013). Amplicons were generated, purified, indexed and sequenced with some modifications according to the Illumina MiSeq 16S Metagenomics Sequence Library Preparation protocol (16S-Metagenomic-library-prep, 2014). An initial PCR reaction contained 25 μL of 2 x KAPA HiFi Hotstart ReadyMix (KAPA Biosystems, Wilmington, MA, United States), 1 μL of forward primer (1 μM), 1 μL of reverse primer (1 μM), 2.5 μL of DNA (∼10 ng/μL) and 20.5 μL of nuclease free H_2_O in a total volume of 50 μL. The PCR reaction was performed on a 96-well Thermocycler using the following program: 95°C for 3 min, followed by 25 cycles of 95°C for 30 s, 55°C for 30 s and 72°C for 30 s and a final extension step at 72°C for 5 min. All amplicons were cleaned using Ampure DNA capture beads (Agencourt-Beckman Coulter; Inc.) following addition of Illumina sequencing adapters and dual-index barcodes to each amplicon with the Nextera-XT Index kit (Illumina Inc., San Diego, CA, United States) according to the manufacturer’s instructions. The amplicon libraries were pooled in equimolar concentrations. The final library was paired-end sequenced at 2 × 300 bp using a MiSeq Reagent Kit v3 on the Illumina MiSeq platform. Sequencing was performed on the Next Generation Sequencing Platform at Teagasc Moorepark research centre, Fermoy, Cork, Ireland.

### Amplicon Data Analysis

16S rRNA gene sequences were analyzed using USEARCH v8 64 bit^1^ (Edgar, 2013) and QIIME, v1.9.0 (Quantitative Insight into Microbial Ecology) (Caporaso et al., 2010), unless otherwise specified the default parameters were used. Paired-end reads were merged using the command fastq_mergepairs in USEARCH by specifying a minimum overlap of 16 bp. Barcode sequences were removed from the merged paired-end sequences using the command extract_barcodes.py in QIIME. We used USEARCH to demultiplex the pre-processed sequencing reads and to generate a quality report. We used the fastq_filter function in USERACH to truncate all the reads to a length of 400 bp and discard sequences shorter than this length and sequences that contained more than four expected base errors per read. The retained high-quality sequencing reads then clustered into operational taxonomic units (OTUs) at 97% sequence identity using the USERACH pipeline. Singletons were discarded from further analysis and the “Gold” reference database^2^ was used to identify and remove chimeras. Taxonomic classification of OTU-representative sequences was performed in QIIME using RDP (Ribosomal Database Project) classifier (Wang et al., 2007) trained against the Greengenes database (DeSantis et al., 2006, release 13_5). Likewise, we used OTU representative sequences to generate a phylogenetic tree in QIIME using ‘muscle’ as alignment method. The generated OTU table, taxonomy information and phylogenetic tree were used to implement the ecological and statistical analyses.

### Statistical Analysis

Due to the intrinsic complexity of our experimental design, contemplating field sampling, we decided to use a dedicated kit for the preparation of soil-derived (i.e., soil and rhizosphere) specimens. This approach appeared not suitable for plant-derived (i.e., root and shoot) specimens. Therefore, the differences in DNA preparation could contribute, at least in part, to apparent differences in the WOSR microbiota composition. For this reason, we generated two independent datasets for the data analysis: one comprising soil-derived microhabitats (soil and rhizosphere samples) and one containing plant-associated microhabitats (root and shoot). Data analysis and visualization were performed using Phyloseq (McMurdie and Holmes, 2013) package from R operated through R Studio v 0.99.893. All OTUs belonging to chloroplast and mitochondria were identified and removed from the data set prior the analysis. To assess differential bacterial abundance between the samples, we used ANCOM (Analysis of Composition of Microbiomes) (Mandal et al., 2015), a statistical test developed for microbial count data, using R with additional parameters multcorr = 2 and sig = 0.05, that is with multiple testing correction at significance 0.05. For alpha diversity analysis, observed OTUs, Chao1 and Shannon indexes, normal distribution of the data were checked with the Shapiro–Walk test. Significant differences in the variance of parameters were evaluated, depending on the distribution of the estimated parameters, either with parametric *t*-test or non-parametric Mann–Whitney–Wilcoxon and Kruskal–Wallis tests to identify significant differences between the two tillage systems and microhabitat zones. *Post hoc* comparison were conducted by Kruskal–Wallis Dunn test. For such analysis, sequencing reads of soil samples (bulk soil and rhizosphere) and plant samples (root and shoot) were rarefied at an even sequencing depth 6,191 and 9,765 reads/sample respectively. To compare community diversity between the samples (beta-diversity), Principal Coordinate Analysis (PCoA) based on Bray-Curtis, sensitive to OTU abundances, and Weighted UniFrac, sensitive to OTU abundances and taxonomic affiliation, distances were calculated by using counts per million transformed OTU abundances. Permutational multivariate analysis of variance using distance matrices was performed in R using the ‘adonis’ function to define the proportion of variance explained by the factors microhabitat and/or tillage. A differential analysis of the OTUs relative abundances using moderated shrinkage estimation for dispersions and fold changes as an input for a pair-wise Wald test was carried out in DESeq2 package from R version 1.14.1 (Love et al., 2014). This test identifies the number of OTUs significantly enriched in different compartments corrected for tillage practices, and in two tillage practices corrected for individual compartment with an adjusted *P*-value (False Discovery Rate, FDR *P* < 0.05). We used a Venn diagram to visualize enriched OTUs, unique and shared, in root and shoot microhabitat zones under CT and ST.

## Results

### General Characterization of the WOSR Microbial Communities

The microbiota of WOSR grown under two cultivation systems, CT and ST, were analyzed at maturity (harvesting stage). 16S rRNA sequencing libraries of the bulk soil, rhizosphere soil, roots and shoots were prepared and sequenced. The analysis generated 992,256 sequence reads of which 691,230 (∼69.64 % total sequence reads) were retained upon quality-filtering. However, these PCR primers were incapable of discriminating between plant-derived (e.g., plastids) and microbial-derived 16S rRNA gene sequences. Therefore, we reasoned that the first step in the data analysis was to identify potential host plant-derived ‘contaminants’ in our dataset (Supplementary Table S2). Indeed, while the plant derived sequences in bulk soil in bulk soil and rhizosphere samples were negligible (below 1%), approximately half of the root and shoot-associated reads were identified as plant derived sequences (Supplementary Table S2 and Figure S1). Upon *in silico* removal of these sequences, we were able to retain enough high quality reads per sample (max = 65,113, min = 6,191, median = 28,801). These sequencing reads were clustered using > 97% sequence similarity to microbial OTUs. The total numbers of microbial OTUs was 2,161 (**Table 1**). Rarefaction curves based Chao1 analysis showed OTU saturation at ∼15,000 sequence reads per sample (Supplementary Figure S2).

**Table 1:** Quality metrics for sequencing data.

Total number of reads and read lengths				
Total number of raw reads before QC	992,256
Total number of assigned reads after QC	691,230
Read length after QC	400 bp

**Assigned reads**	**Bulk Soil**	**Rhizosphere soil**	**Root**	**Shoot**

Average number of reads	29070 ± 12264	32780 ± 20153	42902 ± 16005	60669 ± 1995
Non-target reads (%)	0.25 ± 0.04	0.27 ± 0.10	49.2 ± 5.99	46.56 ± 5.84
Average number of assigned reads	28996 ± 12231	32700 ± 20115	21891 ± 7967	31618 ± 7834
Normalized reads per sample	6191	6191	9765	9765
Average number of assigned OTUs	969 ± 48	962 ± 114	438 ± 115	150 ± 16
Unclassified reads (%)	0.21	0.16	0.09	0.03
% of total useable reads	25.2	28.4	19.0	27.4

### Taxonomic Assemblages of Bacterial Microbiota

**FIGURE 1:**
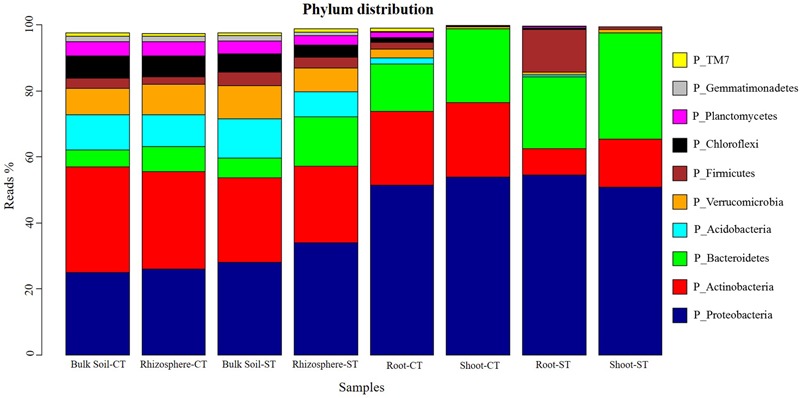
Phylum distribution of the OTUs. Average relative abundance (% of sequencing reads) of 10 most abundant prokaryotic phyla associated with soil, rhizosphere, root and shoot microhabitat zones of WOSR under conventional tillage (CT) and conservation strip tillage (ST), are displayed in different colors. For each sample type, the number of replicates are *n* = 3.

Approximately 99% of WOSR microbiota were represented in the top 10 most abundant bacterial phylum (**Figure 1**). In particular, the phyla *Proteobacteria, Bacteroidetes, Actinobacteria, Acidobacteria, Verrucomicrobia*, and *Chloroflexi* largely dominate the bulk soil and rhizosphere soil microbiota. At the phylum level, bacterial communities of bulk soil and rhizosphere were very similar under CT. However, sequences assigned to phylum *Bacteroidetes* discriminated bulk soil (5.92%) from the rhizosphere (14.92%) profiles under ST. The phylum *Bacteroidetes* was more abundant in shoot communities in both tillage systems which distinguished the shoot from the root microbiota. There was a marked enrichment of the phylum *Firmicutes* (12.93%) and depletion of phylum *Actinobacteria* (7.98%) in root microbiota of ST compared to root under CT (*Firmicutes* 1.91%; *Actinobacteria* 22.40%). The shoot under both tillage practices contained very few microbes assigned to phylum *Firmicutes*. These results highlight a shift in community composition which progressively differentiated the root and shoot bacterial assemblages, from the soil biota; and whose magnitude is influenced, at least in part, by the tillage regime. Moreover, the ANCOM analysis showed that the abundance of 10 bacterial communities at phylum level; *Acidobacteria, Armatimonadetes, Bacteroidetes, Chloroflexi, Firmicutes, Gemmatimonadetes, Nitrospirae, Planctomycetes, Proteobacteria*, and *WS3* were significantly (*P* < 0.05) different in the bulk soil and each compartment under both tillage regimes; CT and ST (Supplementary Figure S3). Our results showed that in WOSR, tillage practice had a marked effect on rhizosphere, root and shoot microbiota but surprisingly, not on bulk soil microbiota. The WOSR bacterial composition at family level showed that, in the bulk soil 77% of the OTUs had less than 1% relative abundance. Families such as *Chthonionbacteraceae* (4%), *Hyphomicrobiaceae* (2.9%), *Bradyrhizobiaceae* (2.8%) and *Bacillaceae* (2.8%) were among the most abundant groups present in the bulk soil (Supplementary Figures S4, S5A). In the rhizosphere samples, 72% of the microbiota were present in abundances of less than 1% of the total population. Here families such as *Sphingomonadaceae* (5%), *Sphingobacteriaceae* (3.5%), *Micrococcaceae* (3.6%) and *Chthoniobacteracaea* (3%) were among the most abundant groups (Supplementary Figure S5B). In the roots of WOSR 39% of the microbiota existed as less than 1% of the total root population. In the roots, *Pseudomonadaceae* were the most abundant family observed, making up 14% of the total OTU count. This was followed by families such as *Sphingobacteriaceae* (9%), *Bacillaceae* (2.8%), *Xanthomonadaceae* (5%) and *Flavobacteriaceae* (4%) (Supplementary Figure S5C). Finally, in the shoots, 23% of the OTUs were present as less 1% of the population. This microhabitat appears to have a very different set of dominant microbes originating from families such a *Sphingobacteriaceae* (12%), *Nocaridiaceae* (9.6%), *Flavobacteriaceae* (8.6%) *Rhizobiaceae* (8%) and *Enterobacteriaceae* (6%) (Supplementary Figure S5D).

### Bacterial Alpha-Diversity and Beta-Diversity

**FIGURE 2:**
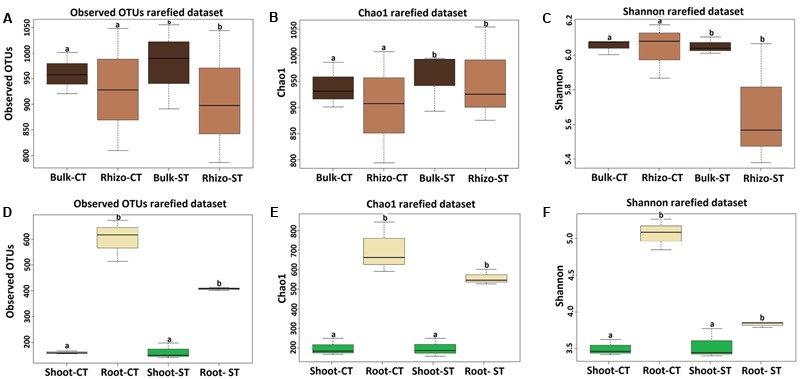
Variation patterns of alpha diversities of the bacterial communities associated with bulk soil, rhizosphere, root and shoot under two tillage practices; CT and ST. The alpha diversity estimates; Total number of observed OTUs, Chao1 estimator and Shannon’s diversity of soil samples (bulk soil and rhizosphere) are displayed in **(A–C)** respectively, and of plant samples (root and shoot) are displayed in **(D–F)** respectively. Sequencing reads of soil samples and plant samples were rarefied at an even sequencing depth 6,191 and 9,765 reads/sample respectively prior the analysis. *Lowercase letters* denote statistically significant differences by Kruskal–Wallis and Dunn’s *post hoc* tests, *P* < 0.05, Benjamini–Hochberg (BH) corrected between the plant compartments within one tillage system. Statistical results of alpha diversity are displayed in Supplementary Excel File WS-1.

We investigated the effect of the tillage and compartment on microbiota composition at the OTU level, which represent the highest taxonomic resolution achievable in our investigation. Alpha diversity, the microbial diversity within each sample, was analyzed based on the OTU richness, Chao1 and Shannon diversity indices (**Figure 2**). To control for differences in sampling effort across microhabitats, we rarefied the soil samples (bulk soil and rhizosphere) to 6,191 and plant samples (root and shoot) to 9,765 reads per sample before calculating the diversity indices. OTU richness was highly dependent on microhabitat type, with high richness values for bulk soil (969 ± 48) and rhizosphere soil (962 ± 114), and consistently decreased in richness estimates in the root samples (438 ± 115) and shoot samples (150 ± 16) (Supplementary Table S4). For diversity and evenness estimates, the soil samples failed to identify a tillage as well as compartmental effects on the WOSR microbiota (*t*-test; Mann–Whitney–Wilcoxon test; Kruskal–Wallis test; *P* > 0.05, **Figures 2A,B,C** and Supplementary Excel File WS-1). On the other side, the plant microhabitats; root and shoot also failed to show a tillage effect (Mann–Whitney–Wilcoxon test; *P* > 0.05, **Figures 2D,E,F** and Supplementary Excel File WS-1). However, there was clear compartment effect observed in the WOSR plant samples (Kruskal–Wallis and Dunn’s *post hoc* tests, *P* < 0.05, Benjamini–Hochberg corrected). The soil samples displayed a greater richness and diversity compared to that of plant samples (**Figure 2**). In particular, the Shannon index showed a marked difference between the root samples of both tillage (CT and ST) practices (**Figure 2F**). Thus, the WOSR microbiota emerged as a progressively gated community whose composition appears largely defined by the plant microhabitat type.

**FIGURE 3:**
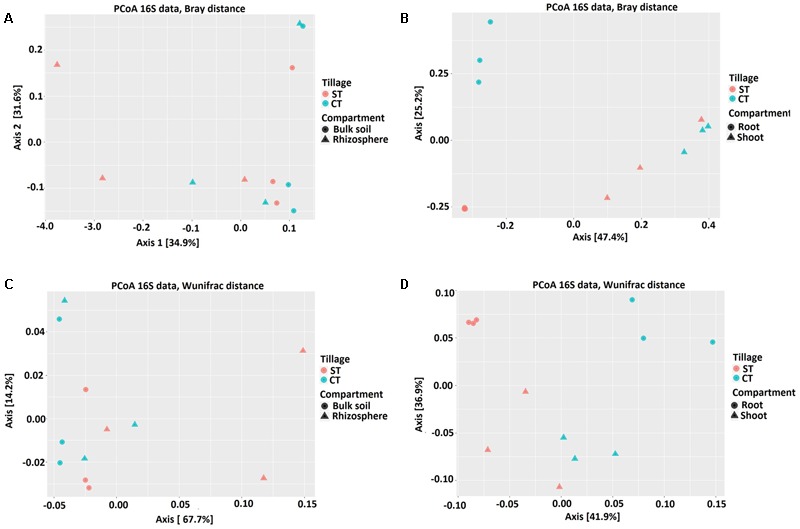
Bacterial community structure of bulk soil, rhizosphere, root and shoot under CT and ST tillage practices. Principal Coordinate Analysis (PCoA) based on Bray-Curtis (BC) and Weighted UniFrac (WUF) distances calculated using counts per million transformed OTU abundances. Comparison between the soil samples; bulk soil and rhizosphere **(A)** BC **(C)** WUF under CT and ST. Comparison between the plant samples; root and shoot **(B)** BC **(D)** WUF under CT and ST. In both panels, colors define the tillage regimes, while shapes depict the indicated compartments. Statistical results of beta diversity are displayed in Supplementary Excel File WS-2.

To elucidate whether the composition of bacterial communities correlated with, the microhabitat and/or tillage system, we used the OTU count data to construct dissimilarity matrices with Bray–Curtis, sensitive to OTUs relative abundance (Bray and Curtis, 1957) and weighted UniFrac, sensitive to OTUs relative abundance and taxonomic relatedness (Lozupone et al., 2011). These matrices were visualized using PCoA as shown in **Figure 3**. At the OTU level, PCoA analyses revealed a clear separation between the root and shoot microhabitats and to a lesser extent between the bulk soil and rhizosphere microbiota. Partitioning of variance (ADONIS) based on Bray–Curtis distance matrix (**Figures 3A,B** and Supplementary Excel File WS-2) of soil samples (bulk soil and rhizosphere) indicated minor contribution of the soil microhabitat type (*P* = 0.05) and showed no influence of tillage practices (*P* > 0.05). However, Weighted UniFrac analysis of the soil samples showed a significant contribution of microhabitat type and tillage methods to the clustering of WOSR soil microbiota. ADONIS based on Bray–Curtis distance matrix and Weighted UniFrac analysis showed that plant microhabitat type (root or shoot), tillage practice, and the their interactions had significant contributions to the differentiation of the root and shoot microbiota (**Figures 3C,D** and Supplementary Excel File WS-2). At the OTU level, bulk soil and rhizosphere bacterial communities share a large degree of similarity. However, when the phylogenetic information was included with OTU relative abundance, a minor separation was observed between the bulk soil and rhizosphere whereas, marked segregation was displayed of the root and shoot samples based on both microhabitat zone and tillage effects (*R*^2^ and *P*-values are listed in **Table 2**). These results further support our hypothesis that the WOSR rhizosphere, root and shoot microbiota are colonized by taxonomically distinct communities, which emerge from the soil biota through progressive differentiation and whose composition is modulated, at least in part, by the tillage practices.

**Table 2:** Statistical analysis of beta diversity.

	Bulk vs. Rhizosphere		Root vs. Shoot	
	*R^2^*	*P*	*R^2^*	*P*
**Bray–Curtis (ADONIS)**				
Compartment	0.12218	0.179	0.17214	0.005^∗∗^
Tillage	0.16541	0.058	0.45064	0.001^∗∗∗^
Tillage and compartment	0.06203	0.584	0.12117	0.018^∗^
**Weighted Unifrac (ADONIS)**				
Compartment	0.20883	0.024^∗^	0.30167	0.001^∗∗∗^
Tillage	0.23882	0.012^∗^	0.33495	0.001^∗∗∗^
Tillage and compartment	0.09149	0.025^∗^	0.13974	0.003^∗∗^

*Significance levels: ^∗^*P* ≤ 0.05; ^∗∗^*P* ≤ 0.01; ^∗∗∗^*P* ≤ 0.001.*

### Differences in the Microbiota of WOSR Microhabitats

**FIGURE 4:**
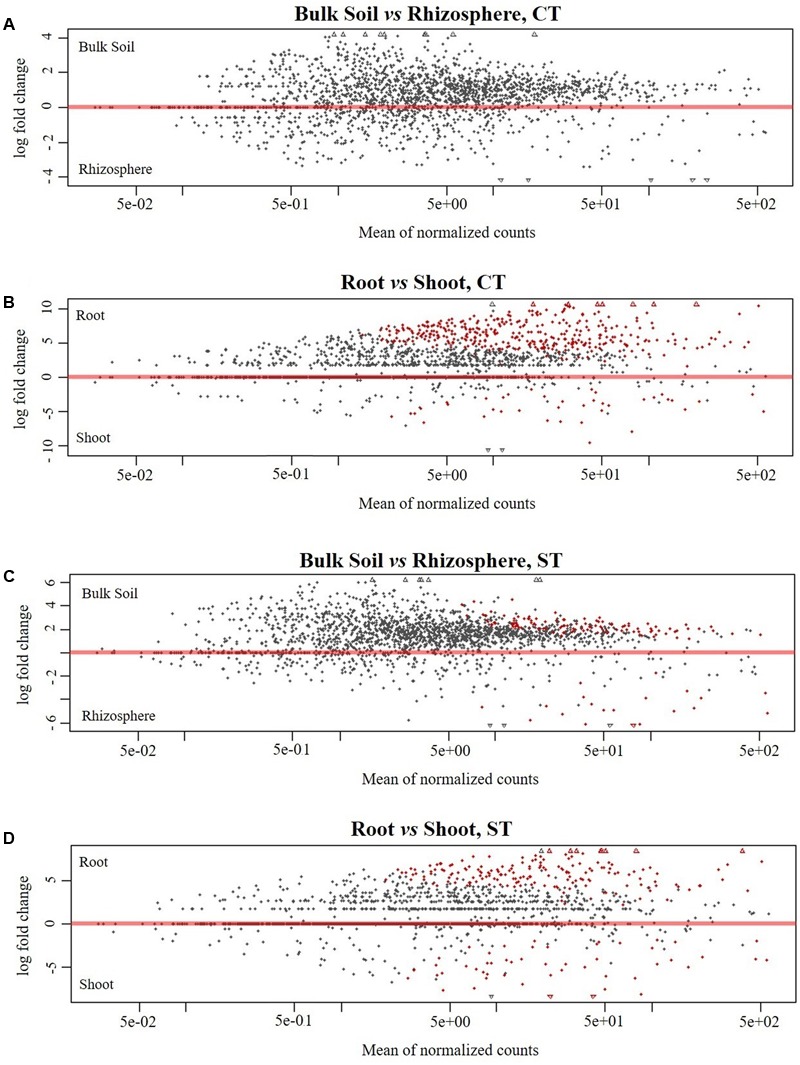
Pair-wise comparisons of the compartments under tillage regimes for enriched OTUs. Comparison of bulk soil and rhizosphere under **(A)** CT **(C)** ST. Comparison of root and shoot under **(B)** CT **(D)** ST. In each plot, the shapes depict individual OTUs whose position on the *x*-axis reflect their abundance (normalized counts) and the position on the *y*-axis the fold change in the indicated comparison. The red color depicts OTUs whose abundance is significantly different in the indicated comparisons (Wald test, *P* < 0.05, FDR corrected). Taxonomy information of significantly enriched OTUs in each compartment under both tillage are displayed in Supplementary Excel File WS3–10.

**FIGURE 5:**
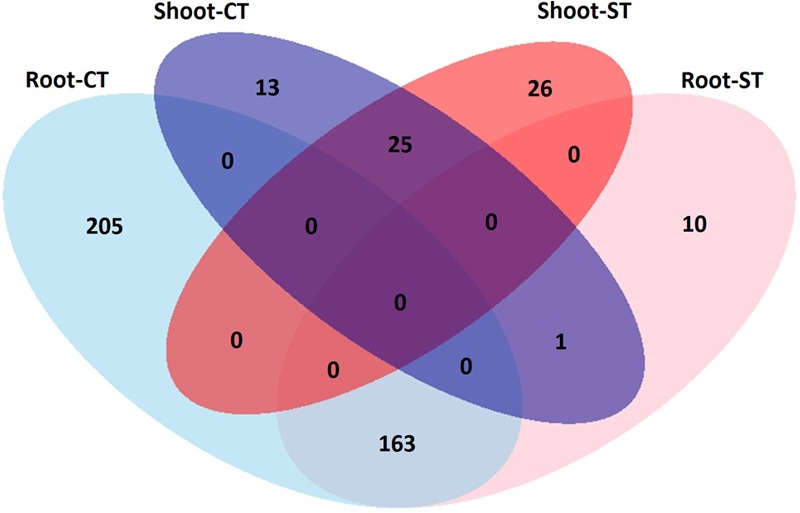
Venn diagram displays the number of OTUs that significantly differentiate root and shoot compartments in the indicated tillage regime; CT and ST (Wald test, *P* < 0.05, FDR corrected).

To identify OTUs which significantly differentiate the bacterial communities in the four microhabitat zones (bulk soil, rhizosphere, root and shoot) and as a result of the tillage regime (CT and ST), we performed a pair-wise comparison using a model based on a negative binomial distribution. This approach shows that the OTUs identified in the bulk soil are progressively excluded from the rhizosphere (**Figures 4A,C** and Supplementary Excel Files WS-3,4,7,8; Walt-test *P* < 0.05, FDR corrected) and in plant samples, the OTUs found in the roots are gradually excluded from the shoot (**Figures 4B,D** and Supplementary Excel Files WS-5,6,9,10; Walt-test *P* < 0.05, FDR corrected) regardless of the tillage regime. Individual bacterial OTUs were enriched in the each microhabitat and contributed to differentiating these communities. Intriguingly, these OTUs represent just a minor fraction of the total WOSR microbiota. For instance, under CT, we observed no significant OTUs enrichment in the rhizosphere compared to bulk soil which suggests that both microhabitat zones share very similar bacterial members. While under ST, 118 and 20 OTUs were enriched in the bulk soil and rhizosphere respectively (**Figure 4D** and Supplementary Excel Files WS-7,8, Wald test, *P* < 0.05, FDR corrected). Our analysis showed that in rhizosphere soil, there was little or no significant enrichment of OTUs as a consequence of the tillage practices used. Whereas in plant samples, 368 and 39 enriched OTUs differentiated root and shoot microhabitats under CT, respectively (**Figure 4B** and Supplementary Excel Files WS-5,6, Wald test, *P* < 0.05, FDR corrected), and 174 and 51 enriched OTUs under ST respectively (**Figure 4D** and Supplementary Excel Files WS-9,10, Wald test, *P* < 0.05, FDR corrected). Thus, the significant enrichment of individual members of the plant habitat bacterial communities represent a distinctive feature of the WOSR root and shoot microbiota. This feature displayed a clear microhabitat zone- and tillage-dependency (**Figure 5** and Supplementary Excel Files WS 11–18). None of the enriched OTUs appeared conserved across the plant microhabitat zones and tillage method and the root and shoot profiles were characterized by distinct patterns. For instance none of the enriched OTUs were shared between the root and shoot in each tillage practice. Moreover, the root profile was characterized with markedly distinct OTU enrichment: 205 OTUs under CT and just 10 under ST.

To further evaluate significantly enriched OTUs as a result of tillage regimes, we performed a similar pair-wise comparison using negative binomial distribution (Supplementary Figure S5 and Table S3, Wald test, *P* < 0.05, FDR corrected). This approach showed that in bulk soil, there was no significant difference in the OTU enrichment between both tillage practices. Under CT there were 5, 13 and 9 enriched OTUs identified in the rhizosphere, root and shoot, respectively. Under ST there were 9, 10 and 1 enriched OTUs identified in the rhizosphere, root and shoot, respectively (Supplementary Table S3).

## Discussion

This study focused on the effect of tillage practices; conventional versus conservational ST, on the microbiota associated with WOSR. The bulk soil bacterial communities under both tillage systems were dominated by *Proteobacteria, Actinobacteria, Acidobacteria* and *Verrucomicrobia* phyla. These phyla typically form a major part of the microbial composition of agricultural soils (Fierer and Jackson, 2006; Montecchia et al., 2015). We found no significant difference in number of OTUs between conventionally tilled soils and in strip–tilled soil. This is in contrast to the findings of Smith et al. (2016) who found that conventionally tilled fields had more OTUs than conservation tilled fields; they also found that bacterial abundance was very low in conventionally tilled soil. We also did not see any significant difference the number of OTUs in the rhizosphere between conventionally tilled soil and strip–tilled soil (Supplementary Figure S6). Although we did observe increases in *Bacteroidetes* in the rhizosphere under ST, there was no major significant difference between the rhizosphere soils and the bulk soils. This would seem to indicate a very limited ‘rhizosphere effect’ in WOSR (i.e., an increased abundance, structural enrichment and diversification of the microbial communities inhabiting the rhizosphere compared to bulk soil). This was further supported by our alpha diversity analysis which showed that the microbiota of the bulk soil and rhizosphere were not distinct from each other. This is in striking contrast with other studies reporting a marked structural differentiation of the rhizosphere profiles from the bulk soil of other annual plants, such as the monocotyledons: barley (Bulgarelli et al., 2015), maize (Peiffer et al., 2013), and rice (Edwards et al., 2015) and an earlier study conducted on OSR using low-resolution profiling techniques (Costa et al., 2006). Our observations are similar to the findings of Bulgarelli et al. (2012) and Lundberg et al. (2012) in *Arabidopsis thaliana* (which is from the same botanical family as OSR) who reported the resemblance of rhizosphere microbiota to the bacterial community of bulk soil samples in multiple soil types. However, our beta diversity Weighted UniFrac analysis showed minor separation between bulk and rhizosphere bacterial communities based on the tillage effect. Again this is in contrast to previous studies which have observed a much more pronounced effect of conventional and conservational tillage practices on soil microbial communities (Carbonetto et al., 2014; Smith et al., 2016; Degrune et al., 2017).

The bacterial communities associated with the roots and shoots of WOSR were found to be dominated by the bacterial phyla *Proteobacteria, Actinobacteria* and *Bacteroidetes*. These three phyla comprised 83–91% of the root microbiota and 98–99% of the shoot microbiota. This is similar to what has been reported for *Arabidopsis thaliana*; (Bulgarelli et al., 2012; Lundberg et al., 2012; Bodenhausen et al., 2013) as well as for other monocotyledons and dicotyledons species (reviewed in Hacquard et al., 2015). Alpha and beta diversity analysis showed pronounced differences in the root and shoot microbiota. There was a clear reduction in OTU number, richness and abundance from the rhizosphere into the root and from the root into the shoot. This observation mirrors the multi-step selection processes proposed for the plant microbiota (Bulgarelli et al., 2013, 2015; Edwards et al., 2015), where a combination of host–microbe and microbe–microbe interactions progressively define the microhabitat zones of the plant microbiota.

When we looked at the effect of tillage practice on the root and shoot microbiota, our alpha diversity analysis suggested that tillage method had little effect on the shoot microbiota. However, alpha diversity indices markedly differentiated root and shoot communities in both CT and ST treatments. This observation was further supported by the PCoA plots of beta diversity which showed pronounced separation of both the root and shoot bacteria communities based on tillage practices. This difference is possibly driven by changes in physical properties of soil such as texture, structure, permeability, nutrient content or pH due to the different tillage methods (as the plant genotype was the same in both treatments, and therefore selective pressure from the plant should be the same in both treatments) (Mathew et al., 2012; Smith et al., 2016).

Degrune et al. (2017) reported a short term temporal change in soil community structure as a result of tillage practice and reported that these changes became less significant at the later growing stages of the plant. Our results are in agreement with this, where at the harvesting stage of WOSR we observed very similar microbiota profiles of soils subjected to conventional and strip-tillage systems. We hypothesize that different tillage practices cause short term changes in the bulk soil microbiota, and although these changes were not lasting in the bulk soil, they are significant enough to affect the initial colonization and community structure of the plant at the germination and seedling stages. This in turn leads to significant and lasting effects on the plant microbiota. These observations prompt further investigation aimed at elucidating the long term impact of tillage practices on the composition of the soil and WSOR microbiota and their ecological services.

Our results showed that the root microbiota appears to be sensitive to tillage practice. This is evidenced by a differential enrichment of individual bacteria likely derived from the soil biota. Are these enriched bacteria a source of plant probiotic functions and what kind of functions can they provide to their host plants? Answering these questions, will bring farmers a step closer to rationally manipulate the plant microbiota through soil tillage management.

## Accession Numbers

The sequences generated in this study are deposited in the European Nucleotide Archive (ENA) under the accession numbers ERX1526681, ERX1526682, ERX1526683, ERX1526684, ERX1526685, ERX1526686, ERX1526687, ERX1526688, ERX1526689, ERX1526690, ERX1526691, ERX1526692, ERX1526693, ERX1526694, ERX1526695, ERX1526696, ERX1526697, ERX1526698, ERX1526699, ERX1526700, ERX1526701, ERX1526702, ERX1526703, and ERX1526704 under the ENA Bioproject PRJEB14407. The scripts used to analyze the sequencing data and generate the figures of this study are available at https://github.com/BulgarelliD-Lab/OSR_microbiota.

## Author Contributions

RR, KG, DD, PF and JS conceived of and designed the experiments. RR performed the experimental work and PC carried out the sequencing work. RR and DB conceived and executed the analysis of the 16S rRNA sequencing dataset. RR, KG, DD, and DB wrote the paper.

## Conflict of Interest Statement

The authors declare that the research was conducted in the absence of any commercial or financial relationships that could be construed as a potential conflict of interest.

## Footnotes

1 http://www.drive5.com

2 http://drive5.com/uchime/gold.fa
