# Supplementary material for: Crop Establishment Practices Are a Driver of the Plant Microbiota in Winter Oilseed Rape (Brassica napus)
Source: Front Microbiol. 2017 Aug 9;8:1489. doi: 10.3389/fmicb.2017.01489 (PMC5553296; doi:10.3389/fmicb.2017.01489)
Supplement: Supplementary file 1 [file Data_Sheet_1.pdf]

Table S1: Physical and chemical characterisation of the soil substrates

Mineral content (g·kg^{-1})	Conventional tillage (CT)	Conservation strip tillage (ST)
Total carbon	2.93	2.95
Total nitrogen	0.27	0.27
Soil organic matter	5.72	5.79
Soil organic carbon	2.52	2.55
C/N ratio	11.93	12.09
pH	6.09	6.15
Classification	Sandy loam	Sandy loam

Table S2: Proportion of reads with and without plant derived from WOSR harvesting stage samples

Sample ID	Compartment type	Reads with plant derived reads	Reads without plant derived reads	Proportion of without plant derived reads (%)
Bn 25	Shoot	73043	38699	52.98
Bn 26	Root	42615	23886	56.05
Bn 27	Rhizosphere	65236	65113	99.81
Bn 28	Bulk soil	48100	47972	99.73
Bn 29	Shoot	69672	35422	50.84
Bn 30	Root	42791	23984	56.05
Bn 31	Rhizosphere	34683	34599	99.76
Bn 32	Bulk soil	32385	32323	99.81
Bn 33	Shoot	87433	38828	44.41
Bn 34	Root	47772	26879	56.27
Bn 35	Rhizosphere	36906	36782	99.66
Bn 36	Bulk soil	15215	15178	99.76
Bn 61	Shoot	32521	18293	56.25
Bn 62	Root	69757	31552	45.23
Bn 63	Rhizosphere	36306	36214	99.75
Bn 64	Bulk soil	39568	39461	99.73
Bn 65	Shoot	55541	30048	54.10
Bn 66	Root	22487	9765	43.43
Bn 67	Rhizosphere	6218	6191	99.57
Bn 68	Bulk soil	14735	14701	99.77
Bn 69	Shoot	45801	28416	62.04
Bn 70	Root	31990	15278	47.76
Bn 71	Rhizosphere	17333	17303	99.83
Bn 72	Bulk soil	24418	24343	99.69
	Total	992526	691230	----
No. of OTUs **with** Plant derived				2182
No. of OTUS **without** Plant derived				2161
Total useful reads in percentage				69.64%

Max, Min, and Mean no. of reads in dataset	
Max	28801.25
Min	65113
Mean	6191

Table S3: Significantly enriched OTUs in comparison to tillage practices (CT vs ST) in bulk soil and plant microhabitat zones

Sample type	Enriched OTUs	Taxonomy of enriched OTUs
Bulk soil - CT	0	-
Bulk soil - ST	0	-
Rhizosphere - CT	5	p__Proteobacteria c__Betaproteobacteria o__Burkholderiales f__Comamonadaceae
		p__Proteobacteria c__Gammaproteobacteria o__Xanthomonadales f__Xanthomonadaceae
		p__Firmicutes c__Bacilli o__Bacillales f__Bacillaceae
		p__Bacteroidetes c__Sphingobacteriia o__Sphingobacteriales f__Sphingobacteriaceae
		p__Bacteroidetes c__Sphingobacteriia o__Sphingobacteriales f__Sphingobacteriaceae
Rhizosphere - ST	9	p__Proteobacteria c__Alphaproteobacteria o__Rhizobiales f__Hyphomicrobiaceae
		p__Proteobacteria c__Alphaproteobacteria o__Rhizobiales f__Hyphomicrobiaceae
		p__Proteobacteria c__Alphaproteobacteria o__Sphingomonadales f__Sphingomonadaceae
		p__Proteobacteria c__Deltaproteobacteria o__Myxococcales f__
		p__Verrucomicrobia c__Opitutae o__Opitutales f__Opitutaceae
		p__Verrucomicrobia c__[Pedosphaerae] o__[Pedosphaerales] f__auto67_4W
		p__Actinobacteria c__Actinobacteria o__Actinomycetales f__Nocardiaceae
		p__Actinobacteria c__Actinobacteria o__Actinomycetales f__Nocardioidaceae
		p__Nitrospirae c__Nitrospira o__Nitrospirales f__Nitrospiraceae
Root - CT	13	p__Proteobacteria c__Alphaproteobacteria o__Rhizobiales f__Hyphomicrobiaceae
		p__Proteobacteria c__Alphaproteobacteria o__Rhizobiales f__Hyphomicrobiaceae
		p__Proteobacteria c__Gammaproteobacteria o__Xanthomonadales f__Xanthomonadaceae
		p__Proteobacteria c__Gammaproteobacteria o__Xanthomonadales f__Xanthomonadaceae
		p__Proteobacteria c__Gammaproteobacteria o__Xanthomonadales f__Sinobacteraceae
		p__Proteobacteria c__Deltaproteobacteria o__Myxococcales f__Haliangiaceae
		p__Proteobacteria c__Deltaproteobacteria o__Myxococcales f__
		p__Chloroflexi c__Ellin6529 o__ f__
		p__Actinobacteria c__Actinobacteria o__Actinomycetales f__Thermomonosporaceae

		p__Actinobacteria c__Actinobacteria o__Actinomycetales f__Micromonosporaceae
		p__Actinobacteria c__Actinobacteria o__Actinomycetales f__Nocardiaceae
		p__Actinobacteria c__Actinobacteria o__Actinomycetales f__Sanguibacteraceae
		p__Actinobacteria c__Actinobacteria o__Actinomycetales f__Thermomonosporaceae
Root - ST	10	p__Proteobacteria c__Betaproteobacteria o__Burkholderiales f__Comamonadaceae
		p__Proteobacteria c__Betaproteobacteria o__Burkholderiales f__Comamonadaceae
		p__Proteobacteria c__Gammaproteobacteria o__Enterobacteriales f__Enterobacteriaceae
		p__Proteobacteria c__Gammaproteobacteria o__Pseudomonadales f__Pseudomonadaceae
		p__Proteobacteria c__Alphaproteobacteria o__Caulobacterales f__Caulobacteraceae
		p__Firmicutes c__Bacilli o__Bacillales f__Bacillaceae
		p__Firmicutes c__Bacilli o__Bacillales f__Bacillaceae
		p__Bacteroidetes c__Flavobacteriia o__Flavobacteriales f__Flavobacteriaceae
		p__Bacteroidetes c__Flavobacteriia o__Flavobacteriales f__[Weeksellaceae]
		p__Bacteroidetes c__Sphingobacteriia o__Sphingobacteriales f__Sphingobacteriaceae
Shoot - CT	9	p__Proteobacteria c__Betaproteobacteria o__Methylophilales f__Methylophilaceae
		p__Proteobacteria c__Betaproteobacteria o__Burkholderiales f__Comamonadaceae
		p__Proteobacteria c__Alphaproteobacteria o__Rhizobiales f__Brucellaceae
		p__Firmicutes c__Bacilli o__Bacillales f__Paenibacillaceae
		p__Bacteroidetes c__Flavobacteriia o__Flavobacteriales f__[Weeksellaceae]
		p__Bacteroidetes c__[Saprospirae] o__[Saprospirales] f__Chitinophagaceae
		p__Actinobacteria c__Actinobacteria o__Actinomycetales f__Nocardiaceae
		p__Actinobacteria c__Actinobacteria o__Actinomycetales f__Streptomycetaceae
Shoot - ST	1	p__TM7 c__TM7-3 o__ f__

*Right, number of significantly enriched OTUs retrieved from a moderated estimation of fold change and pair-wise Wald test (FDR, $P < 0.05$) between tillage practices (CT vs ST) of WOSR prokaryotic microbiota profiles. Left, taxonomy of frequently enriched OTUs. (CT) = conventional tillage, (ST) = conservation strip tillage, (p) = phylum, (c) = class, (o) = order, (f) = family

Table S4: Overview of OTU data for the microbiota of WOSR at harvesting stage

	Complete Microbiome (Average No. of OTUs per sample)	Core Microbiome (No. of OTUs common to all six samples)	Dominant OTUs in Core Microbiome (No. of common OTUs ≥1% relative abundance)
Bulk soil	969 ± 48 (899-1036)	375	15 [8-12]
Rhizosphere	926 ± 114 (800-1068)	292	22 [14-17]
Root	438 ± 115 (334-577)	143	21 [15]
Shoot	150 ± 16 (133-180)	68	25 [15]

Data is based on the rarefied data set. Numbers in () brackets represents the ranges. Numbers in [] represent the number of taxa represented at the family level.

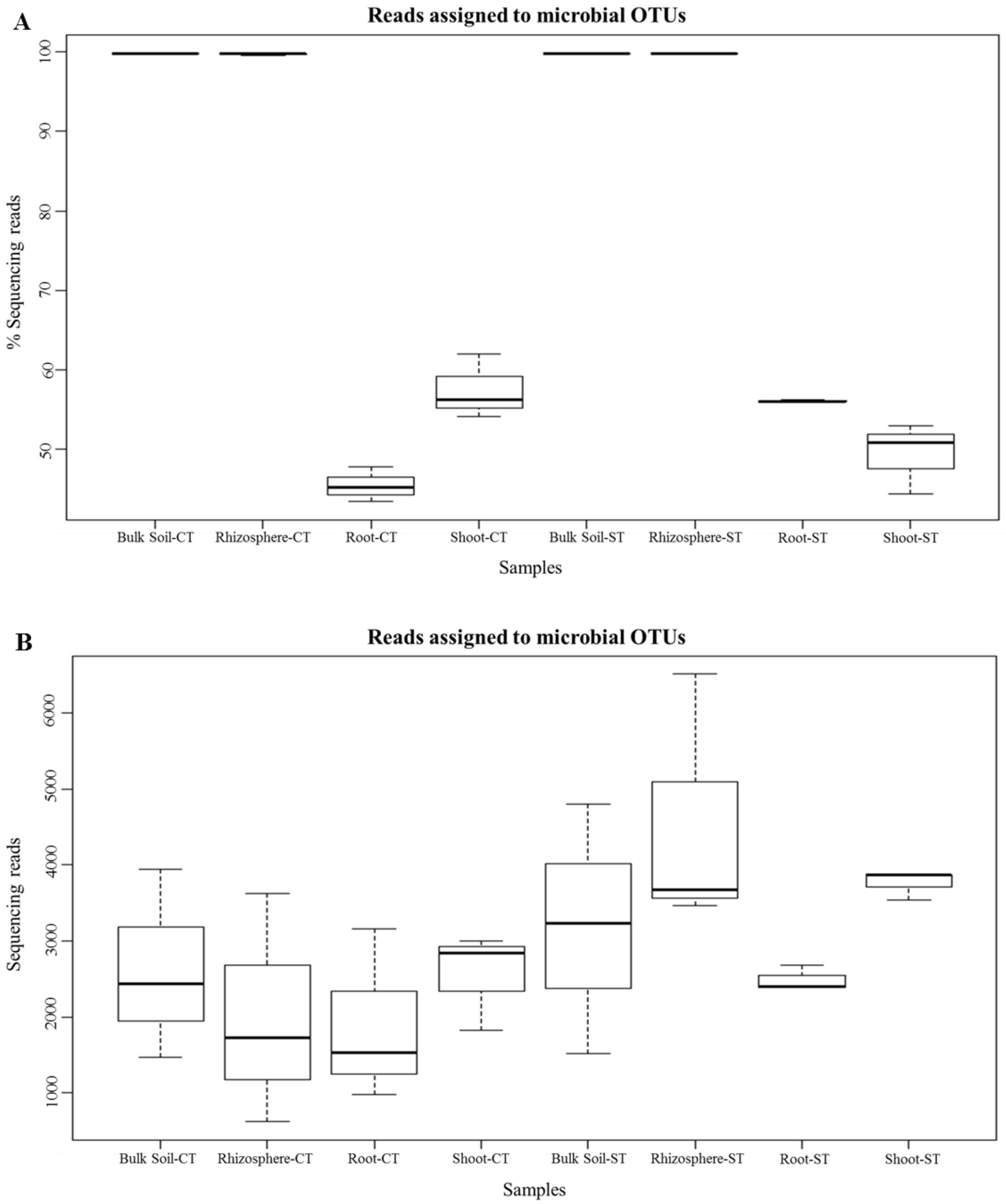

Figure S1: (A) Proportion of microbial-derived OTUs across compartments. X axis refers the sample type and Y axis refers the % reads assigned to microbial- derived OTUs. (B) Number of average reads per sample type. X axis refers the sample type and Y axis refers the number of reads assigned to microbial-derived OTUs.

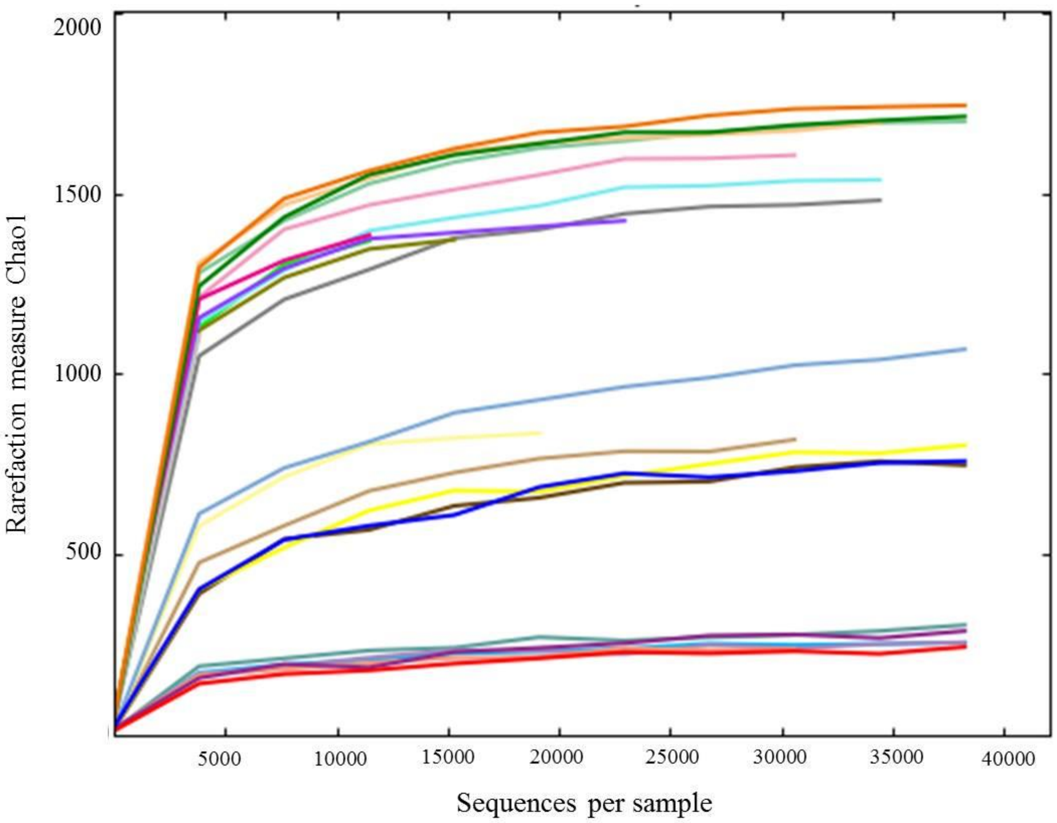

Figure S2: Rarefaction curves of individual samples from WOSR, generated in qiime.

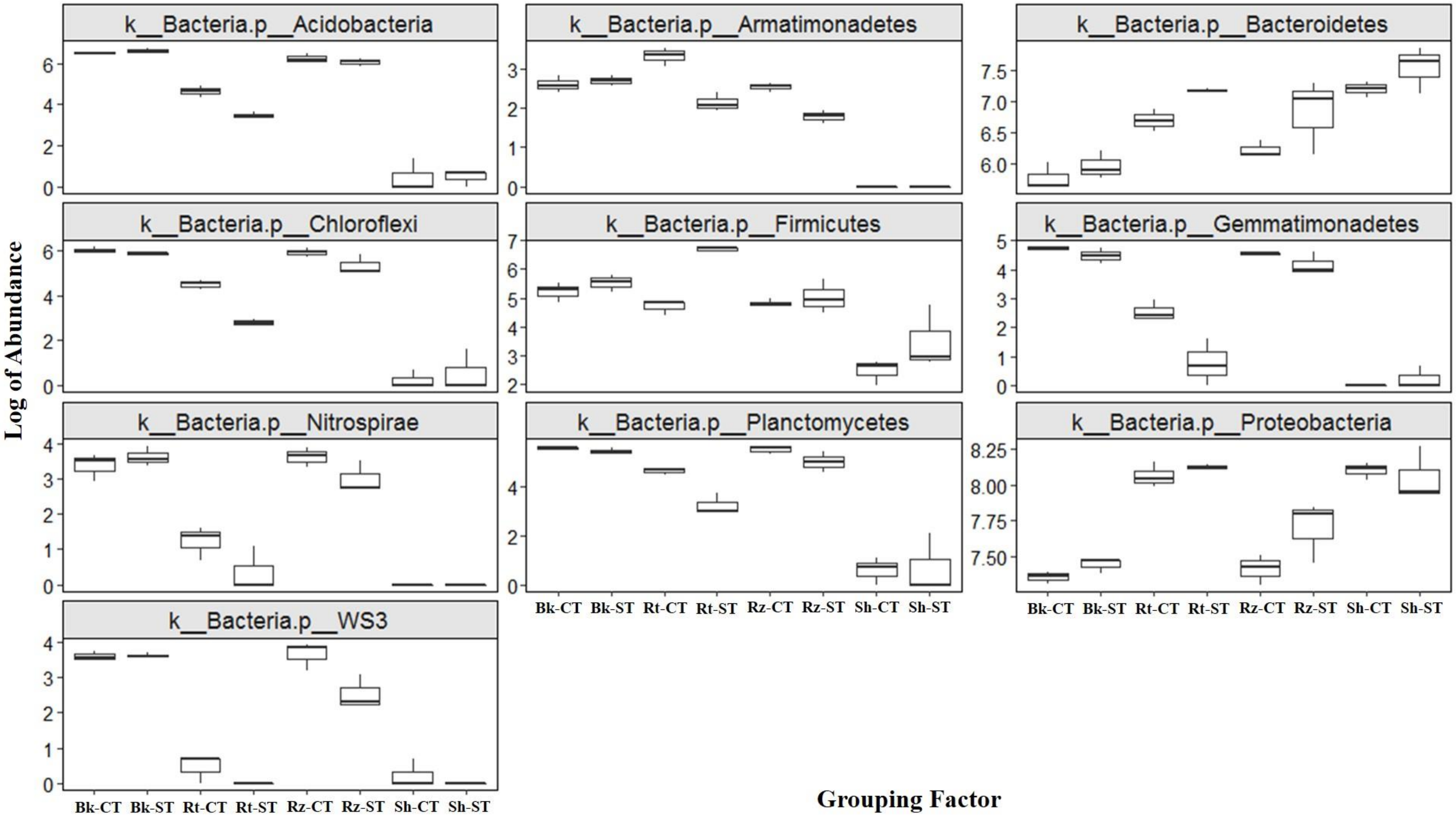

Figure S3: Differential composition of WOSR bacterial microbiota at phylum level (ANCOM, $P < 0.05$, FDR correcte

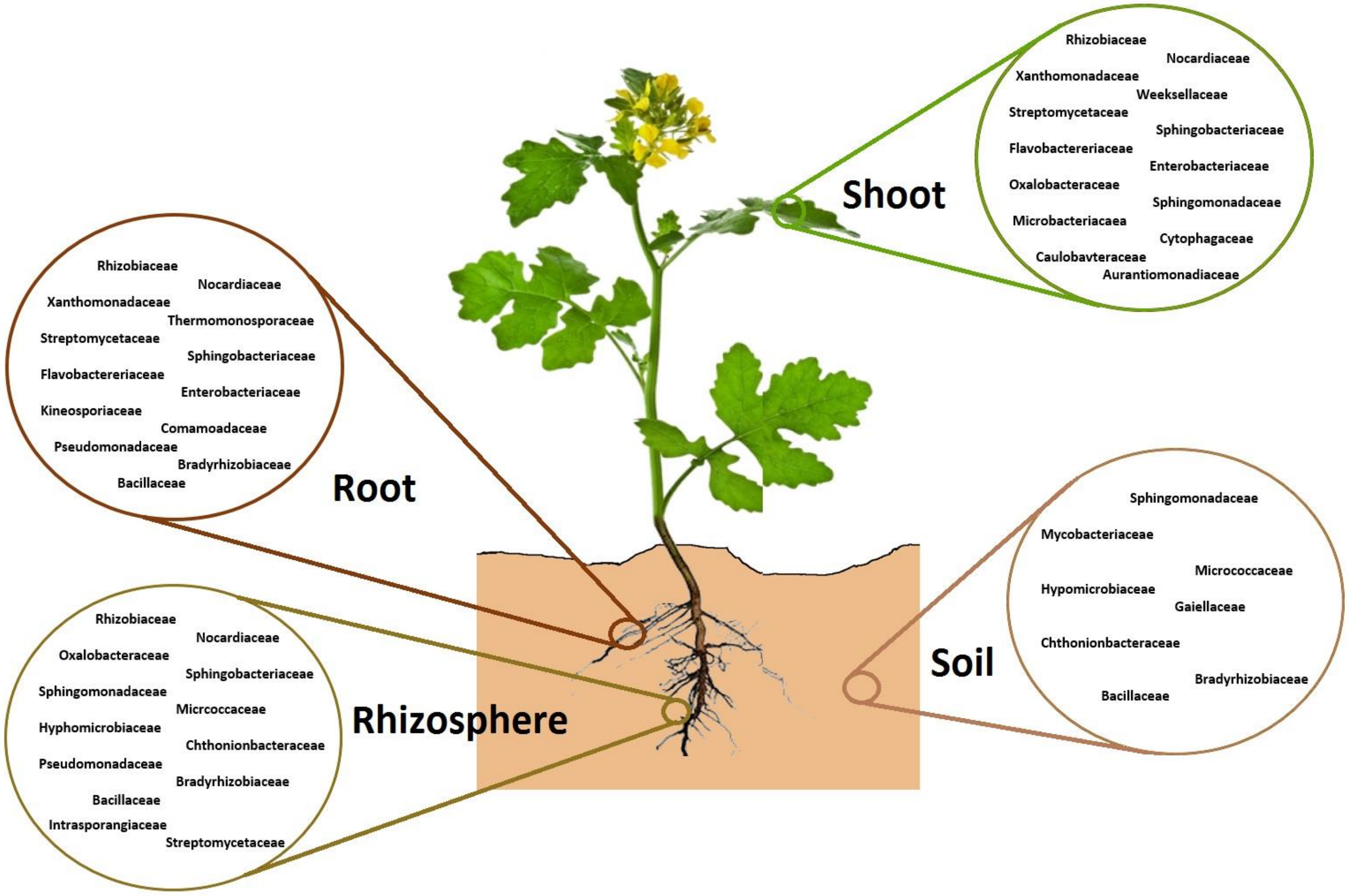

Figure S4: The conserved dominant microbiome of winter Oilseed Rape (*Brassica napus* var. compass) at harvesting stage. The conserved dominant microbiome is defined as those OTUs found in all six samples of a microhabitat, at greater than 1% relative abundance (e.g. the core shoot microbiome are those OTUs found in all six samples of shoot material at greater than 1% relative abundance)

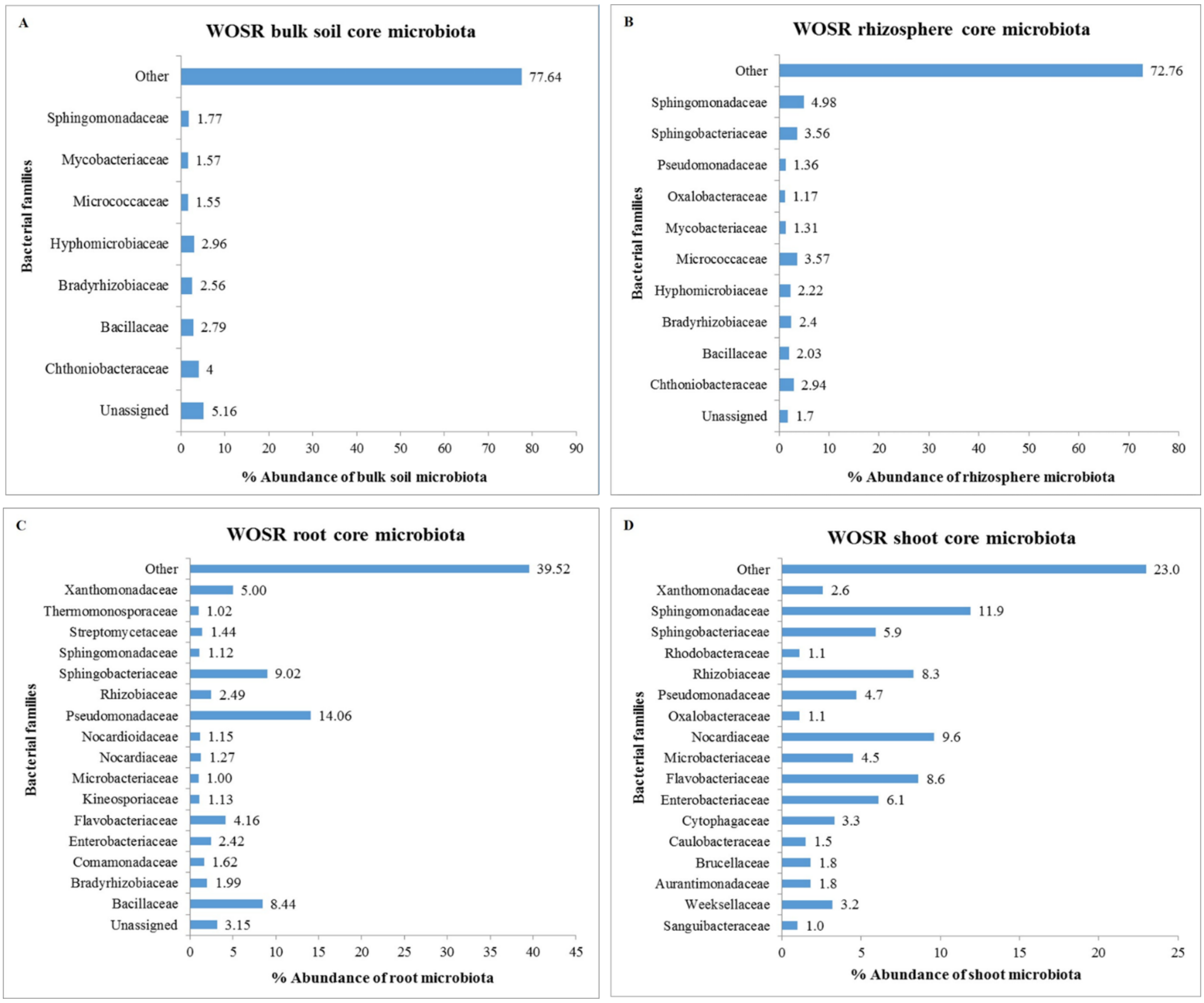

Figure S5: The conserved dominant microbiota of the WOSR in (A) bulk soil, (B) rhizosphere (C), root and (D) shoot compartments at harvesting stage based on the rarefied data set.

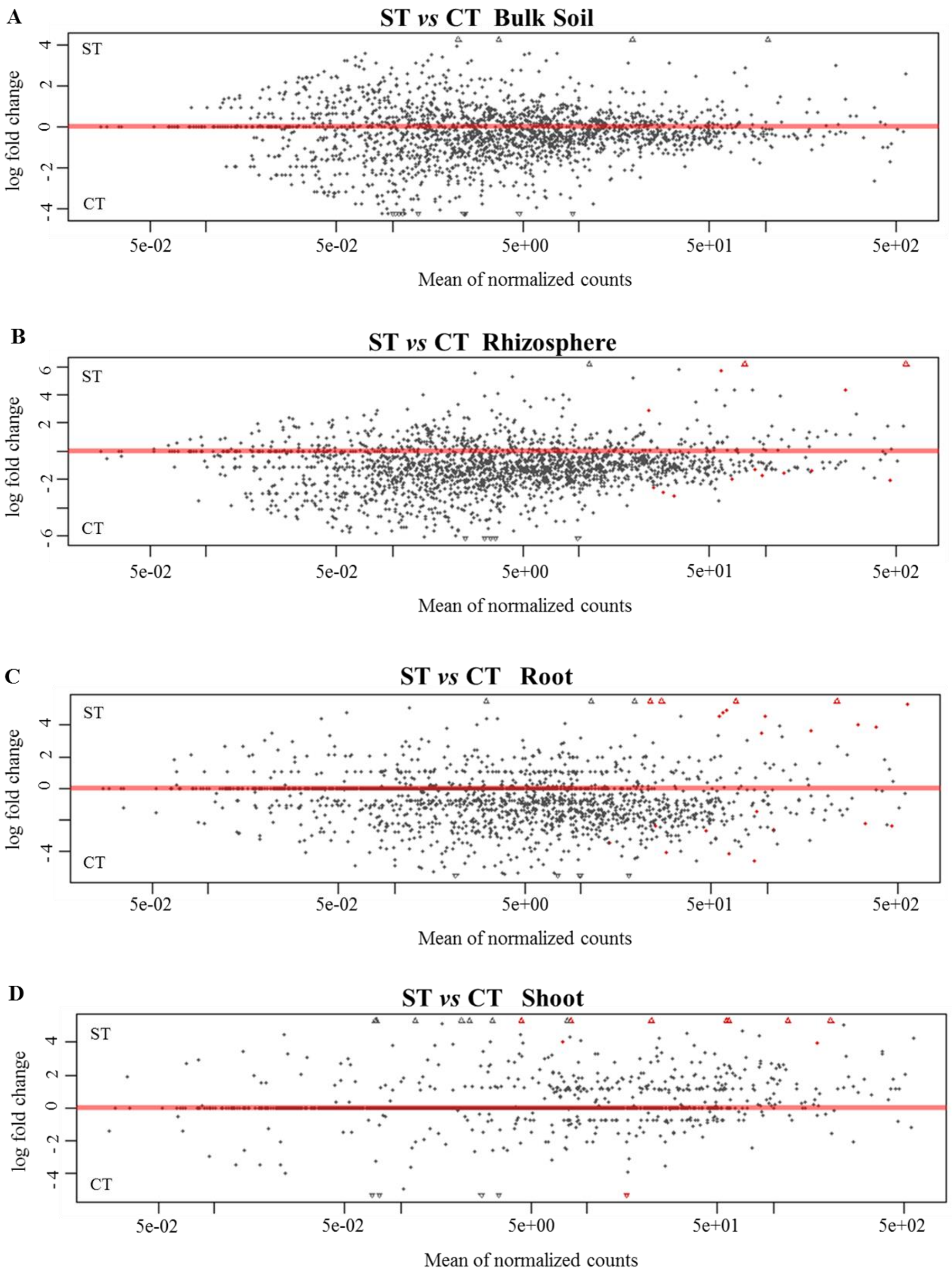

Figure S6: Pair-wise comparisons of the tillage practices; CT and ST under individual compartments; (A) bulk soil, (B) rhizosphere, (C) root and (D) shoot for enriched OTUs. In each plot, the shapes depict individual OTUs whose position on the x-axis reflect their abundance (normalised counts) and the position on the y-axis the fold change in the indicated comparison. The red colour depicts OTUs whose abundance is significantly different in the indicated comparisons (Wald test, $P < 0.05$, FDR corrected).
